# Prevalence of gastrointestinal worms in Wonosobo and thin-tailed sheep on the slope of Mount Sumbing, Central Java, Indonesia

**DOI:** 10.14202/vetworld.2019.1866-1871

**Published:** 2019-11-27

**Authors:** Zein Ahmad Baihaqi, Irkham Widiyono, Wisnu Nurcahyo

**Affiliations:** 1 Student of Postgraduate Program of Veterinary Science, Faculty of Veterinary Medicine, Universitas Gadjah Mada, Yogyakarta, Indonesia; 2 Department of Internal Veterinary Medicine, Faculty of Veterinary Medicine, Universitas Gadjah Mada, Yogyakarta, Indonesia; 3 Department of Parasitology, Faculty of Veterinary Medicine, Universitas Gadjah Mada, Yogyakarta, Indonesia

**Keywords:** gastrointestinal, nematodes, prevalence, sheep, Wonosobo sheep

## Abstract

**Aim:**

This study was conducted to determine the prevalence of gastrointestinal (GI) worms in Wonosobo and thin-tailed sheep from the slope of Mount Sumbing.

**Materials and Methods:**

Fecal samples (n=305) were collected directly from the rectum of Wonosobo and thin-tailed sheep during the dry and rainy seasons in Wonosobo Regency, Central Java Province, Indonesia. The presence of GI helminth eggs in the fecal samples was assessed using the modified McMaster egg counting technique. The identification of the eggs or oocysts was done on the basis of their morphology and size.

**Results:**

The highest prevalence of GI worms was observed in male thin-tailed sheep (76.47%) during the rainy season, whereas the lowest prevalence was observed in female Wonosobo sheep (47.36%) during the dry season. The types of GI nematodes observed in these two types of sheep were *Haemonchus contortus*, *Ostertagia* spp., *Trichostrongylus* spp., *Bunostomum*spp., *Trichuris* spp., and *Moniezia* spp. The GI worms with the highest prevalence were of *Haemonchus* spp. and were observed in male thin-tailed sheep. The prevalences of the two types of sheep assessed at an altitude of 1150 m above sea level were higher than those observed at the altitude of 1586 m. The prevalence of clinical and sub-clinical parasites infestation in Wonosobo and thin-tailed sheep in Kwadungan village was significantly different (p<0.05).

**Conclusion:**

This study showed that two types of local sheep on the slope of Mount Sumbing are infected with various GI worms during the dry and rainy seasons. The highest prevalence of GI worms was found in thin-tailed sheep at an altitude of 1150 m above sea level during the rainy season, with *H*. *contortus* being the most prevalent GI parasites.

## Introduction

Gastrointestinal (GI) nematodiasis infections are a major problem in small ruminants. These infections may lead to diseases, leading to death or decreased production of milk, meat, and wool [1]. Sheep are highly susceptible to GI nematode infections [2]. GI nematode infections have been widely reported to affect ruminants, causing various adverse effects in these animals, such as weight loss and death. Consequently, such parasitic infections significantly impact the incomes of farmers [3]. Parasite infections in small ruminants are a serious problem in developing countries, especially due to poor environment, nutrition, and sanitation. GI nematode infections are one of the major health problems that hamper livestock production worldwide [4]. Parasite infections cause losses, such as morbidity, mortality, reduction in feed conversion ratio, poor quality of meat, and higher costs to control parasites [2]. The parasites that often infect small ruminants are Family *Trichostrongylidae* (*Trichostrongylus* spp., *Ostertagia* spp., *Cooperia* spp., and *Haemonchus contortus*), Family *Strongylidae* (*Oesophagostomum* spp., *Strongyloides* spp., and *Chabertia ovina*), and Family *Ancylostomatidae* (*Bunostomum* spp.) [5]. GI nematode infection is a major problem in small ruminant husbandry worldwide due to the impact of infection on the productivity and cost of the livestock industry. In fact, nematode parasitism is often detected in developing countries due to unbalanced nutritional resources, which disrupt natural immunity, thereby predisposing the host animal to further infections, lowering the productivity, and increasing the mortality of the host [6].

Kwadungan and Butuh villages reside on the slopes of Mount Sumbing at altitudes of 1150 and 1586 m above sea level, respectively. Kwadungan village is located below Butuh village. These two villages abundantly raise Wonosobo and thin-tailed sheep. A study on slaughterhouses in Supela, Bhilai, and India reported that the seasonal prevalence of GI parasitic infections is higher during the rainy season (94.60%) than in the summer (87.50%) and winter (63.15%) [7]. Environmental conditions, particularly temperature and humidity, affect the distribution of species and the presence of parasites [8]. Research in Aceh Province found that the level of GI parasite infections in highland and lowland livestock was 22% and 66.6%, respectively. This means that the infections in the lowlands are higher than those in highlands. Temperature and humidity greatly influence the survival of parasites [9]. Parasitic diseases in an area are affected by topographic and geographic factors as well as the temperature and humidity levels [10]. The key factors which may affect nematode population include humidity and vegetation [11]. A study in Kashmir showed that male sheep had a higher prevalence of family *Strongyle*, *Strongyloides*, *Eimeria* and Genus *Nematodirus*, and *Moniezia* spp. than female sheep [12]. Wonosobo Regency is 520 km away from Jakarta. It is located in the middle of Java Island and classified as highlands with an altitude reaching 2250 above sea level. This region is located at 7^0^.11’.20” to 7^0^’.24” South Latitude, and 109^0^.44’.08” to 110^0^.04’.32” West Longitude, with a tropical climate divided into two seasons (rainy and dry seasons). Wonosobo Regency has been breeding Wonosobo sheep for generations. This mongrel sheep was developed in 1957 by crossbreeding Texel sheep and thin-tailed or fat-tailed sheep, and it has been raised in Wonosobo Regency, Central Java Province, since then. Thin-tailed sheep is a local Indonesian sheep with a relatively small body, thin wool, medium body length, and thin tail [13].

The environmental conditions in tropical regions constitute an ideal habitat for parasitic species, especially in areas with a wet climate [14]. Moisture and limited sunlight facilitate parasite egg survival [15]. GI parasites infections spread in tropical countries because the environmental conditions are very suitable for the transmission of parasites [16]. The presence of parasites infections in Wonosobo and thin-tailed sheep in Kwadungan and Butuh villages requires in-depth studies during the dry and rainy seasons because these villages are located in a highland (on the slope of Mount Sumbing), where the temperature and humidity allow the growth of GI parasites.

This study aimed to reveal the health status of two types of local Indonesian sheep on the slope of Mount Sumbing in relation to GI parasites infections so that the government can make appropriate policies for the welfare of the farmers in these regions.

## Materials and Methods

### Ethical approval

This research was approved by the Institutional Ethical Committee, Faculty of Veterinary Medicine, Universitas Gadjah Mada, Yogyakarta, Indonesia. Number: 0013/EC-FKH/Int./2019.

### Study area

This study was conducted in Butuh and Kwadungan villages located on the slopes of Mount Sumbing (1586 and 1150 m above sea level, respectively), Wonosobo Regency, Central Java, Indonesia. Kwadungan village has an average temperature of 23-25°C with a humidity of 81%, and Butuh village has a temperature of 16-20°C with a humidity of 73%.

### Study design and sampling

This study used 305 sheep from the farms belonging to the community on the slope of Mount Sumbing. Fecal samples were collected from 1.5 to 2-year-old sheep from Butuh village (65 Wonosobo sheep and 85 thin-tailed sheep) and Kwadungan village (83 Wonosobo sheep and 72 thin-tailed sheep). Sampling was carried out during the dry season (September 2018-November 2018) and the rainy season (February 2019-March 2019). These two types of sheep had been intensively bred and forest-grass-fed. The average number of sheep per cage was 2-3.

### Collection and examination of fecal samples

Fecal samples were collected from the rectum; a composite was made and ~2 g of feces were placed in a labeled zip-lock plastic bag and stored at 4°C. The parasitological examination was carried out at BBVet (Indonesian Veterinary Center) in Wates, Special Region of Yogyakarta. The calculation of the number of eggs per gram (EPG) was performed according to the nematode egg counts, modified McMaster technique with fecal flotation solution use sugar solution [17]. Singh *et al*. (2013) classified sheep tested positive on fecal examination into two: Subclinical (EPG <1600) and clinical (EPG >1600).

### Statistical analysis

The GI nematodiasis parasites prevalence results were analyzed by Chi-square using SPSS version 20.0 (IBM Corp., NY, USA).

## Results

The prevalence of GI parasites during the rainy season was higher than that during the dry season, and their prevalence in male sheep was higher than that in the females (Table-1). The highest and lowest preva­lence were detected during the rainy season in male thin-tailed sheep (76.47%) and during the dry season in female Wonosobo sheep (47.36%), respectively (Table-1).

**Table-1:** Prevalence of gastrointestinal nematodiasis parasites in sheep.

Type of sheep	Male sheep		Female sheep		Total		Χ^2^-value	p-value
		
	n	Positive (%)	n	Positive (%)	n	Positive		
(September-November 2018)								
Wonosobo sheep	32	18 (56.25)	38	18 (47.36)	70	36 (51.43)	1.347	0.246
Thin-tailed sheep	35	25 (71.42)	33	21 (63.63)	68	46 (67.65)		
(February-March 2019)								
Wonosobo sheep	31	19 (61.29)	47	27 (57.44)	78	46 (58.97)		
Thin-tailed sheep	34	26 (76.47)	55	38 (69.09)	89	64 (71.91)		
Total	132	88 (66.67)	173	104 (60.14)	305	192 (62.95)		
χ^2^-value	1.378							
p-value	0.240							

Table-2 shows the six types of parasites found in Wonosobo and thin-tailed sheep. The most prevalent GI parasites infections in both types of sheep were caused by *H. contortus*, and the lest prevalent ones were caused by *Bunostomum* spp. and *Trichuris* spp. in Wonosobo and thin-tailed sheep, respectively.

**Table-2:** Correlation between sheep genetics and gastrointestinal nematodiasis parasites.

GI parasites	Wonosobo sheep (n=148)	Prevalence (%)	Thin-tailed sheep (n=157)	Prevalence (%)
	
	Positive		Positive	
Ostertagia spp.	65	43.91	59	37.57
Trichostrongylus spp.	60	40.54	67	42.69
Trichuris spp.	21	14.18	17	10.82
Bunostomum spp.	18	12.16	25	15.92
Haemonchus spp.	73	49.32	87	55.41
Moniezia spp.	32	21.62	34	21.65

GI=Gastrointestinal

Table-3 shows that the average prevalence of GI parasites infections in the thin-tailed sheep was higher (67.05%) than that in Wonosobo sheep (52.30%) in Butuh village. The prevalences of sub-clinical and clinical GI parasite infections in thin-tailed sheep were 56.47% and 10.58%, respectively. On the other hand, the prevalences of sub-clinical and clinical infections in Wonosobo sheep were 43.07% and 9.23%, respectively. In addition, Table-4 shows that the average prevalence of parasite infections in thin-tailed sheep in Kwadungan village was also higher (73.61%) than the average prevalence in Wonosobo sheep (57.83%). The prevalences of sub-clinical and clinical infections in thin-tailed sheep were 48.61% and 25%, respectively, whereas the prevalences of sub-clinical and clinical infections in Wonosobo sheep were 44.57% and 13.25%, respectively.

**Table-3:** Prevalence of clinical and sub-clinical gastrointestinal nematodiasis parasites infestation in Wonosobo and thin-tailed sheep in Butuh village.

Location	Sheep (n)	Prevalence		Overall prevalence	χ^2^-value	p-value

		Sub-clinical	Clinical			
Butuh (1.586 mdpl)	W (65)	43.07% (28)	9.23% (6)	52.30% (34)	3.359	0.067
	T (85)	56.47% (48)	10.58% (9)	67.05% (57)		

W=Wonosobo sheep, T=Thin-tailed sheep, Sub-clinical <1600, Clinical EPG >1600. EPG=Eggs per gram

**Table-4:** Prevalence of clinical and sub-clinical gastrointestinal nematodiasis parasites infestation in Wonosobo and thin-tailed sheep in Kwadungan village.

Location	Sheep (n)	Prevalence		Overall prevalence	χ^2^-value	p-value

		Sub-clinical	Clinical			
Kwadungan (1.150 mdpl)	W (83)	44.57% (37)	13.25% (11)	57.83% (48)	5.051*	0.025
	T (72)	48.61% (35)	25% (18)	73.61% (53)		

W=Wonosobo sheep, T=Thin-tailed sheep, Sub-clinical <1600, Clinical EPG >1600. *Indicates values varying significantly at p<0.05. EPG=Eggs per gram

## Discussion

This study was conducted on 305 Wonosobo and thin-tailed sheep (male and female), which showed that the prevalence of GI parasite infections during the rainy season was higher than that during the dry season. The factors that lead to a higher prevalence during the rainy season are related to climatic conditions, such as humidity and temperature, which support the growth and development of parasites, thus increasing the number of infective larvae during the rainy season [16].

The prevalence in the males of both types of sheep was higher than that in the females, which is in line with the result of a previous report [17]. Furthermore, the results presented here corroborate with those of a previous study, which reported that the prevalence of GI parasites in male sheep (75.6%) in the Himalayas was higher than that in the females (44.8%) [18]. Female sheep tend to be more resistant to infection after puberty due to the effect of estrogen stimulation on their immune response [19]. Various GI parasite infections exert serious effects on the health and fertility of sheep. Parasites can affect all the age groups and both sexes [20]. Internal parasites go out of control and cause damage when their number increases above the level the animals can tolerate [21].

The parasites found in this study were *Ostertagia* spp., *Trichostrongylus* spp., *Bunostomum* spp., *Trichuris* spp*., H*. *contortus*, and *Moniezia* spp. Abebe and Esayas reported the presence of GI nematodes, such as *H. contortus*, *Trichostrongylus* spp., *Oesophagostomum* spp., *Bunostomum* spp., *Strongyloides* spp., *Cooperia* spp., *Nematodirus*, and *Trichuris* in various regions [22]. *Haemonchus* spp. was found to be the most prevalent GI nematode in the study presented here, agreeing with the results reported in the previous studies [23-26]; however, *Ostertagia* spp. and *Trichostrongylus* spp. had lower prevalence. Female *H. contortus* parasites are fertile, being able to produce 10,000 eggs every day [26]. This plays a significant role in the high prevalence of *H. contortus* nematode infections in Wonosobo Regency. The rate of GI parasite infections in sheep in several countries varies. The dominant GI nematode infection in Garole sheep is *H*. *contortus* (63.91%), followed by *Oesophagostomum* spp. (16.25%), *Trichostrongylus* spp. (10.50%), and *Strongyloides* spp. (4.16%) [27]. *H. contortus* often infects sheep and goats, and the main target organ is abomasum. The female parasites can produce 10,000 eggs a day. The larvae develop through animal feces and then mature in nature through forage. *H. contortus* nematodes may hamper the infected animal’s fertility, growth, and appetite and cause anemia, edema, and even death [18]. They feed on blood, and thus they are highly pathogenic, causing a significant economic loss in small ruminant husbandry [28]. The symptoms of *Haemonchosis* are pale skin and loss of plasma protein, causing submaxillary edema [29]. The factors that influence the prevalence of GI nematodes are economic conditions and education level of the farmer, grazing management standards, and anthelmintics used [30]. The overall incidence of higher nematode infections in the investigated regions can be attributed to the decreased immunity of the host due to malnutrition [31]. Malnutrition also increases the vulnerability of animals to parasitic infections. Poor animal health also reduces livestock resistance to the symptoms of infections [32].

Wonosobo sheep is a crossbred between Texel sheep and thin-tailed sheep (Indonesian local sheep). Such crossbreeding process is expected to result in a good meat yield because of the Texel sheep genetics and confers resistance to diseases and adaptation to tropical environments because of the thin-tailed sheep genetics [13]. Based on the observation conducted in the two villages during the rainy and dry seasons, GI parasite infections in thin-tailed sheep were higher than those in Wonosobo sheep in the two villages. The infection rate in the rainy season was observed to be higher in this study than that in the dry season, agreeing with the results reported by Velusamy *et al*. [33], Das *et al*. [34], Shakya *et al*. [35], Kanojiya *et al*. [36], Gul and Hidayatullah [37], and Dixit *et al*. [38]. Given that Wonosobo sheep have been raised in Indonesia since 1957, it is expected that they have adapted to the region. A study in Ireland showed that Texel sheep are typically less susceptible to nematodes than Suffolk sheep [39].

The prevalence of GI parasites in Kwadungan village (1150 m above sea level) in the two types of sheep (Wonosobo and thin-tailed) was higher than that in Butuh village (1586 m above sea level), and this observation is in line with the results of a previous report [40]. In fact, these two villages implemented the same breeding management. The rate of GI parasite infections in cattle aged > 12 months at an altitude of 800-1200 m above sea level was 12%, whereas the rate at an altitude of 0-200 m was 78% [9]. Kwadungan village had a higher temperature and humidity than Butuh village, and these climatic conditions are believed to support the growth of GI parasites larvae. The prevalence of GI nematodes varies among regions, depending on the climatic conditions and the management practices implemented [12]. Parasite infections in small ruminants are greatly affected by forage contaminated with parasites larvae [30].

## Conclusion

This study shows that two types of local sheep on the slope of Mount Sumbing at an altitude of 1150-1586 m above sea level are infected with GI parasites, but the level of infection is affected by the altitude. Moreover, the prevalence of GI parasites infections in thin-tailed sheep is higher than that in Wonosobo sheep. The prevalence of the infections is lower at the altitude of 1560 m above sea level than that at the altitude of 1150 m. The prevalence in male sheep is higher than that in female sheep. The dominant type of GI parasites is *Haemonchus* spp.

## Authors’ Contributions

ZAB, IW, and WN designed the study. ZAB conducted the field survey. ZAB, IW, and WN collected samples and examined them in the laboratory. All authors drafted and revised the manuscript. All authors have read and approved the final manuscript.
